# Association between decreased p53 expression, elevated serum CagA levels, and oral squamous cell carcinoma

**DOI:** 10.1016/j.clinsp.2025.100632

**Published:** 2025-04-02

**Authors:** Minxiao Lin, Jing Wang, Xiaowu Yao

**Affiliations:** a Department of Stomatology, the Second Affiliated Hospital of Shantou University Medical College, Guangdong, PR China; b Department of Otolaryngology, the Second Affiliated Hospital of Shantou University Medical College, Guangdong, PR China

**Keywords:** OSCC, p53, Helicobacter pylori, CagA

## Abstract

**Objective:**

p53 is a key tumor suppressor, aCnd loss of p53 function promotes the progression of many cancers. *Helicobacter Pylori* (HP) is mainly involved in the progression of gastric carcinoma, but its role in Oral Squamous Cell Carcinoma (OSCC) is controversial. The primary objectives of this study were to investigate the expression levels of p53 in OSCC tissues and to examine the serum levels of CagA in OSCC patients. Additionally, the authors aimed to explore the potential association between p53 expression and CagA levels in OSCC.

**Method:**

A total of 65 patients diagnosed with OSCC and 42 healthy volunteers were recruited in this study. The clinical pathological parameters of all patients were collected. Reverse transcription-quantitative polymerase chain reaction was performed to detect the expression of p53 in tissues. Receiver Operating Characteristics Curve (ROC) analysis was used to assess the sensitivity of p53 for the diagnosis of OSCC. The concentration of Cytotoxin-Associated gene A (CagA) in serum was assessed by enzyme-linked immuno sorbent assay.

**Results:**

The results indicated that the p53 expression in oral mucosal tissues was downregulated while the concentration of CagA in serum was increased in OSCC patients. Besides, p53 expression was correlated with tumor stage. OSCC patients showed a higher HP positive rate than in healthy people.

**Conclusions:**

In conclusion, this study demonstrated that decreased p53 expression and elevated serum CagA levels might be correlated with OSCC progression and diagnosis.

## Highlights

• In OSCC patients, p53 expression in oral mucosal tissues was downregulated.
• The concentration of CagA in the serum of OSCC patients was increased.
• A correlation was observed between p53 expression and the tumor stage in OSCC.
• The decreased p53 expression may be related to the progression of OSCC.
• Elevated serum CagA levels could potentially serve as a factor for OSCC diagnosis.

**Synopsis**

• The present study found that the expression of p53 was suppressed in the CagA-positive group, suggesting that decreased p53 expression and elevated serum CagA levels might be correlated with OSCC progression and diagnosis.

• The present study demonstrated the role of p53 and HP in OSCC, which helps develop novel therapeutic strategies to treat OSCC.

Alt-text: Unlabelled box

## Introduction

According to the Global Cancer Statistics 2018, oral cancer accounts for about 2 % of all new cases of cancers.^1^ Oral Squamous Cell Carcinoma (OSCC) is the most common oral malignancy, accounting for approximately 90 % of oral cancers, and its increasing incidence in recent years has created a heavy global health burden.^2^ OSCC involves multiple areas of the mouth, including the palate, buccal mucosa, floor of the mouth, tongue, and alveolar ridge.^3^ Long-term smoking, alcoholism, betel nut chewing, unbalanced diet, oral infection, disturbed oral microbiome, and human papillomavirus are all important causes of OSCC.^4^, ^5^, ^6^, ^7^ An interesting study has found that consuming cranberries and blueberries can be effective in preventing OSCC.^6^ For the treatment of OSCC, surgery, radiotherapy, and chemotherapy are the current strategies. However, the side effects of these treatments still exist, such as salivary gland hypofunction, spinal accessory nerve injury, and tumor metastasis and recurrence, which bring great pain to the body and spirit of patients.^8^, ^9^, ^10^ Therefore, it is necessary to further explore the pathogenesis of OSCC.

*Helicobacter Pylori* (HP) is a gram-negative, spiral-shaped microaerophilic bacterium that colonizes the gastric epithelium and causes bacterial infection.^11^ The presence of HP is dangerous, and patients infected with HP may develop gastritis, stomach ulcers, and even develop gastric carcinoma.^12^ CagA, the main virulence factor of HP, encodes the CagA protein in the pathogenic island of Cag and is up-regulated in a variety of gastric diseases.^13^ A previous study shows that HP-induced heparinase promotes the colonization of HP, which triggers gastritis.^14^ In addition, HP-induced endoplasmic network stress and release of pro-inflammatory mediators increase the risk of gastric carcinoma.^15^ Whereas, the destruction of the gastric environment enhances the interaction between the mouth and the gut microbes.^16^ The oral cavity is considered the extragastric reservoir of HP.^17^ A previous review indicates that HP in the oral cavity leads to the progression of chronic periodontitis and is associated with a variety of oral diseases.^18^ However, the biological function of HP in OSCC is rarely studied. p53 is a classic tumor suppressor gene whose expression is suppressed in various cancers.^19^ In this study, the authors aimed to explore the expression of p53 and the role of HP in OSCC, which might provide a theoretical basis for the treatment of OSCC.

## Methods and materials

### Clinical trial and sample collection

These Clinical Trials follow the CONSORT Statement rules. The study included 65 patients diagnosed with OSCC and 42 healthy volunteers. The complete clinical data of OSCC patients, including sex, age, tumor stage, degree of differentiation, negative or positive lymphatic metastasis, and whether they were smokers or drinkers were collected in Table 1. The primary tumors were located in the tongue, gingiva, buccal mucosa, and other sites. All OSCC patients had no detectable serious systemic diseases (such as communicable diseases). Besides, all OSCC patients had not been treated for OSCC or taken antibiotics for at least two weeks prior to sampling. All subjects consented to clinical examination and sampling. Written informed consent was obtained from all subjects. Fasting blood samples were obtained from all study participants by venous cannula, loaded into vials supplemented without anticoagulant, and placed at room temperature for coagulation for 15 min. Then, the blood samples were centrifuged at 1000× *g* for 15 min to obtain serum samples and then stored at −20 °C for subsequent analysis. In addition, the oral mucosa tissue specimens (3 × 3 × 1 mm) were aseptically collected from all 107 subjects transferred into a sterilized microcentrifuge tube, and stored at −80 °C for further analysis.

**Table 1:** The relationship between the expression of p53 in OSCC and clinical pathological parameters of patients.

Characteristics	n	p53		p-value
		High (*n* = 32)	Low (*n* = 33)	
Sex				0.7216
Female	38	18	20	
Male	27	14	13	
Age (years)				0.1478
< 60	41	23	18	
≥ 60	24	9	15	
T stage				0.0256
I–II	49	28	21	
III–IV	16	4	12	
Differentiation				0.0916
High	25	9	16	
Low	40	23	17	
Lymphatic metastasis				0.2358
Negative	47	21	26	
Positive	18	11	7	
Smoker				0.5351
No	45	21	24	
Yes	20	11	9	
Drinker				0.2558
No	36	20	16	
Yes	29	12	17	

OSCC, Oral Squamous Cell Carcinoma.

### RNA isolation and reverse transcription-quantitative polymerase chain reaction (RT-qPCR)

Total RNA from the oral mucosa tissue specimens was extracted using TRIzol reagent (Vazyme, Nanjing, China) according to the manufacturer's instructions. The quality and concentration of RNA were detected by a NanoDrop spectrophotometer (Thermo Fisher Scientific, Waltham, MA, USA). The Evo M-MLV RT Premix for qPCR (Accurate Biotechnology Co., Ltd, Changsha, China) kit was used for reverse transcription. qPCR was performed using the SYBR Green Premix Pro Taq HS qPCR Kit (Accurate) with the thermocycling conditions: 95 °C for 30 s, 40 cycles of 95 °C for 5 s, and 60 °C for 30 s, and a dissociation stage. The specific primer was synthesized by Tsingke Biotechnology Co., Ltd. (Beijing, China) and listed as follows: p53, forward, 5′-GTGGAAGGAAATTTGCGTGT-3′ and reverse, 5′-AGCTGTTCCGTCCCAGTAGA-3′; Glyceraldehyde-3-Phosphate Dehydrogenase (GAPDH), forward 5′-GAAGGTGAAGGTCGGAGTC-3′ and reverse 5′-GAGATGGTGATGGGATTTC-3′. Each sample in each experiment was performed in triplicate. The relative expression levels of related genes were quantified and compared to the internal control GAPDH and analyzed using the 2^−ΔΔCT^ method.

### HP positive detection

Firstly, bacterial genomic DNA was extracted using the Life PureLink Genomic DNA Kit (Thermo Fisher) and stored at −20 °C for further analysis. After that, qPCR was performed using the HP nucleic acid kit obtained from Bioesn Biotechnology Co., Ltd (Shanghai, China) according to the instructions. The thermocycling conditions were 95 °C for 3 min, 40 cycles of 95 °C for 10 s and 60 °C for 30 s, and a melting curve stage. Finally, the positive or negative HP was determined according to the Cycle Threshold (CT) value of the detection channel (CT ≤ 35, positive; no CT value, negative).

### Enzyme-linked immuno sorbent assay (ELISA)

The expression of CagA in serum samples was detected by a specific ELISA kit (Enzyme-linked Biotechnology Co., Ltd, Shanghai, China) according to the instructions. The OD value of each well was detected by the microplate reader (Thermo Fisher). The obtained results were normalized against the total protein concentration in each sample for intersample comparison.

### Statistical analysis

The SPSS 21.0 software was used to analyze data. Data are expressed as mean ± Standard Deviation (SD). Student's *t*-test was used for comparison between the two groups. Statistical analyses were performed using GraphPad Prism software (v8.0.1, GraphPad Software Inc., San Diego, *CA*, USA). The potential diagnostic value of p53 in OSCC was presented by Receiver Operating Characteristics (ROC) curve analysis; *p* < 0.05 indicates that the difference is statistically significant.

## Results

### Decreased expression of p53 in OSCC tissues

**Fig. 1:**
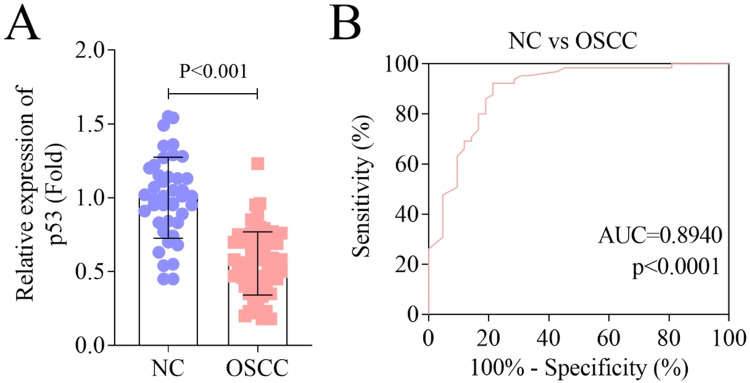
Decreased expression of p53 in OSCC tissues. (A) RT-qPCR was used to detect the expression of p53 in NC and OSCC groups; (B) ROC curves of p53 in OSCC. RT-qPCR, Reverse Transcription-quantitative Polymerase Chain Reaction; OSCC, Oral Squamous Cell Carcinoma; NC, Normal Control.

p53 is considered a biomarker in many cancers.^20^^,^^21^ In order to explore the role of p53 in OSCC, the authors detected the expression of p53 in OSCC patients and healthy volunteers. The results indicated that p53 expression was downregulated in OSCC oral mucosa tissue specimens (Fig. 1A). Moreover, as indicated in Table 1, 65 patients were divided into high-level (*n* = 32) and low-level (*n* = 33) according to the expression of p53, further analysis demonstrated that the p53 level was independent of age, sex, differentiation degree, lymphatic metastasis, and whether the patient was a smoker or a drinker, but correlated with tumor stage. In addition, the ROC curve showed that the Area Under the ROC Curve (AUC) of p53 in oral mucosa tissues was 0.8940, implying the potential diagnostic value of p53 in OSCC (Fig. 1B).

### Increased serum levels of CagA in OSCC patients

**Fig. 2:**
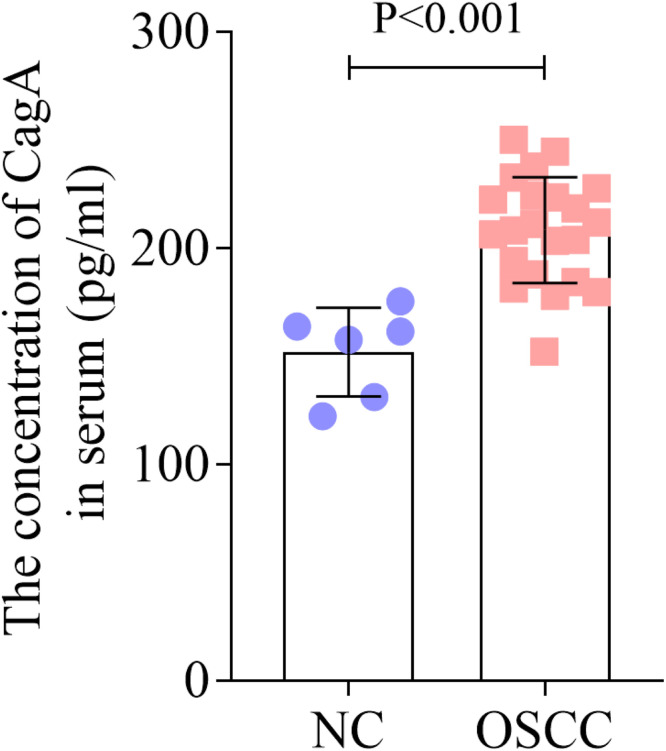
Increased serum level of CagA in OSCC patients. The concentration of CagA in serum in NC and OSCC groups was analyzed by ELISA. CagA, Cytotoxin-Associated gene A; OSCC, Oral Squamous Cell Carcinoma; ELISA, Enzyme Linked Immuno Sorbent Assay; NC, Normal Control.

To explore the role of HP in OSCC, the authors detected the concentration of CagA in serum. The results illustrated that the concentration of CagA in serum was increased in OSCC patients (Fig. 2). Additionally, Table 2 showed that HP nucleic acid positive rate was significantly higher in OSCC patients compared with the healthy people.

**Table 2:** Comparison of HP nucleic acid positive rates between oral cancer group and normal oral mucosa group detected by qPCR method.

Group	n	HP(+)	HP(-)	p-value
OSCC	65	22	43	0.0246
NC	42	6	36	

HP, *Helicobacter Pylori*; qPCR, quantitative Polymerase Chain Reaction.

### Decreased expression of p53 in CagA positive patients

**Fig. 3:**
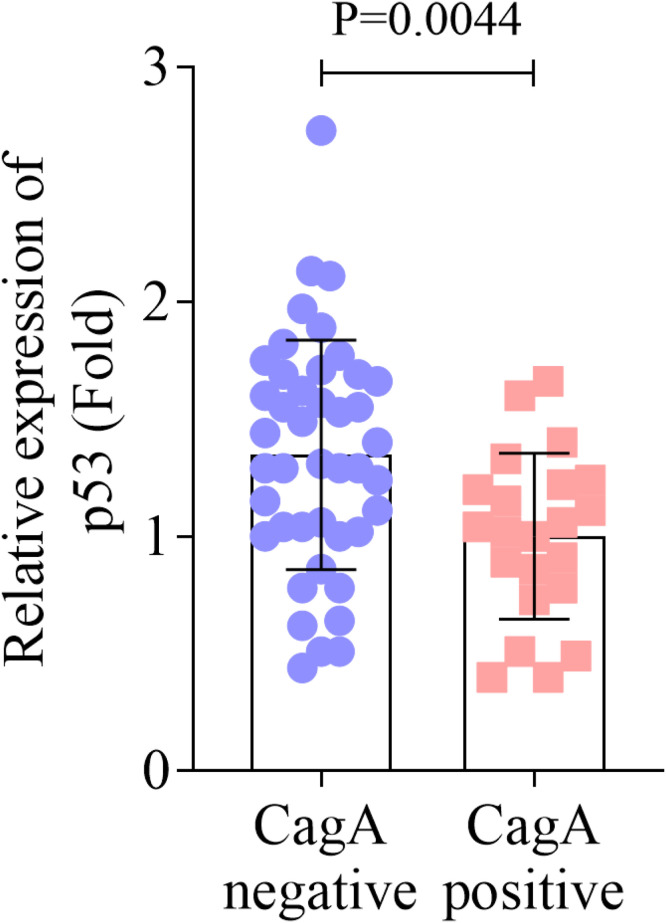
Decreased expression of p53 in CagA positive patients. The expression of p53 in CagA positive and negative groups was assessed by RT-qPCR assay. CagA, Cytotoxin-Associated gene A; RT-qPCR, Reverse Transcription-quantitative Polymerase Chain Reaction.

Based on the results of CagA concentration in serum, OSCC patients were divided into two groups: CagA negative (*n* = 43) and CagA positive (*n* = 22). The qPCR results showed that the expression of p53 in oral mucosa tissues was decreased in CagA positive patients compared with the CagA negative patients (Fig. 3).

## Discussion

p53 is an important transcription factor, that can regulate the expression of multiple target genes and participate in the regulation of various physiological and pathological activities, such as metabolism, aging, DNA repair, cell cycle arrest, and cell death.^22^ More importantly, dysfunction of p53 function usually occurs in the progression of most human malignancies. Therefore, p53 is an effective target for cancer suppression.^23^ In this study, the expression of p53 was decreased in OSCC tissues, which was consistent with previous research.^24^^,^^25^ Besides, ROC results indicated that p53 has potential diagnostic value in OSCC. Similarly, p53 always regarded as a biomarker for diagnosis and prognosis in different diseases.^26^^,^^27^ In addition, the clinical pathological parameters results illustrated that the high or low expression of p53 was correlated with the tumor stage of OSCC.

HP infection is serious and tricky. HP induces multiple genetic changes during adhesion and colonization, expresses multiple virulence factors (such as CagA), and triggers multiple adaptive mechanisms, causing chronic inflammation and tissue damage of the gastric mucosa.^28^ HP can be divided into CagA-positive and CagA-negative strains,^29^ Worldwide, about 60 % of HP infections are caused by CagA-positive strains. The increased CagA expression is associated with a variety of cancer phenotypes, such as sustained proliferation, tumor cell infiltration, invasion, and migration.^30^ In the present study, the authors found that the concentration of CagA in serum was upregulated in OSCC. Besides, HP positive rate was higher in OSCC patients. In fact, the effect of HP on OSCC is debatable. A previous study demonstrates that HP is an unlikely contributing factor for OSCC pathogenesis in contrast to gastric cancer.^31^ Another more recent study finds the presence of HP in histological sections of OSCC and concludes that HP may be a risk factor for developing oral lesions such as oral cancer,^32^ which is consistent with these results. The different conclusions may be due to the improved testing techniques, geographical and ethnic differences of patients, and changes in dietary habits. Additionally, the present study found that the expression of p53 was suppressed in the CagA-positive group, these findings highlight the potential diagnostic value of p53 and CagA in OSCC, but further studies are needed to establish any causal relationships.

In conclusion, this study suggested the potential diagnostic value of p53 and HP in OSCC, which helps develop novel therapeutic strategies to treat OSCC. However, this study has the defects of a small sample size, a single source, and lack of *in vivo* and *in vitro* studies results, which will be further remedied in future studies.

## Conflicts of interest

The authors declare no conflicts of interest.

## Data Availability

The datasets used and/or analyzed during the current study are available from the corresponding author upon reasonable request.
